# Expanding the phenotypic spectrum of Beaulieu–Boycott–Innes syndrome: A case report of a novel THOC6 gene mutation associated with ambiguous genitalia and disorders of sexual development

**DOI:** 10.1097/MD.0000000000043621

**Published:** 2025-08-01

**Authors:** Mousa Humeedat, Maaweya Jabareen, Baraa Maraqa, Husein Sarahneh, Nedal Manasra

**Affiliations:** a Faculty of Medicine, Palestine Polytechnic University, Hebron, Palestine; b Pediatric department, Palestine Red Crescentic Society, Hebron, Palestine; c Faculty of Medicine, College of Medicine, Hebron University, Hebron, Palestine.

**Keywords:** ambiguous genitalia, anorectal malformation, BBIS, THOC6

## Abstract

**Rationale:**

Beaulieu–Boycott–Innes syndrome (BBIS) is a rare autosomal recessive neurodevelopmental disorder caused by THOC6 gene mutations. While BBIS typically presents with intellectual disability, dysmorphic features, and congenital anomalies, its association with ambiguous genitalia and disorders of sexual development has not been previously reported. Expanding the phenotypic spectrum is crucial for early diagnosis and management.

**Patient concerns:**

A 35-day-old Palestinian infant presented with loud breathing sounds and cough. Examination revealed dysmorphic facial features, ambiguous genitalia, and anorectal malformation. Further evaluation identified bilateral inguinal gonads and congenital heart defects, including a ventricular septal defect, patent foramen ovale, and peripheral pulmonary stenosis.

**Diagnoses:**

Chromosomal analysis showed a normal male karyotype (46, XY). Whole exome sequencing identified a novel homozygous splice site mutation in the THOC6 gene (c.155+1G>T), confirming the diagnosis of BBIS. Both parents were found to be heterozygous carriers.

**Interventions:**

The patient underwent surgical correction of the anorectal malformation and received supportive care for respiratory infection. A multidisciplinary follow-up plan was initiated involving pediatrics, endocrinology, neurology, cardiology, and genetics.

**Outcomes:**

Postsurgical recovery was uneventful, but the patient exhibited growth retardation and developmental delay. Long-term monitoring is ongoing to assess neurodevelopmental progress and organ function.

**Lessons:**

This case reports a novel presentation of BBIS associated with ambiguous genitalia, emphasizing the importance of considering THOC6 mutations in patients with syndromic features and congenital anomalies. Early genetic testing is vital for diagnosis and management planning.

**Key Points**

- Beaulieu–Boycott–Innes syndrome (BBIS) is a rare neurodevelopmental disorder resulting from a THOC6 gene mutation.
- (c.155+1G>T) is a novel variant associated with BBIS.
- BBIS syndrome is associated with a wide range of clinical features.

## 1. Introduction

Beaulieu–Boycott–Innes syndrome (BBIS) is a rare autosomal recessive neurodevelopmental condition linked to the THO complex subunit 6 (THOC6).^[1]^ As of early 2020, 7 years after the syndrome was first described and its molecular basis identified, only 19 affected individuals have been reported in the literature.^[2]^ THOC6 is an integral component of the THO/transcription/export (TREX) complex, which plays a crucial role in messenger ribonucleic acid (mRNA) transcription, processing, and the export of spliced mRNA.^[3]^

The TREX complex, a core component preserved across species, is deeply embedded in the workings of the cell, ensuring its smooth operation and balance. It plays a pivotal role in the creation of mRNA and a plethora of other cellular processes. Its existence is imperative for the accurate transcription, processing, and export of genetic messages while also serving as a guardian against deoxyribonucleic acid (DNA) harm and upholding the distinct identities of cells during both embryonic development and in specialized adult tissues.^[4]^

Pühringer and Hohmann et al used cryo-electron microscopy to unveil the first 3D structure of the human TREX complex core. They found that it comprises 7 proteins, forming a large complex with 4 copies of each. This structure suggests TREX can bind to mRNA and its proteins in different ways, potentially aiding in selecting fully modified mRNA for export. Additionally, their work sheds light on how mutations in TREX can lead to diseases like BBIS.^[5]^

In 2013, Beaulieu et al discovered that homozygous mutations in THOC6 were linked for the first time to Beaulieu–Boycott–Innes syndrome (BBIS, OMIM 613680), characterized by syndromic autosomal recessive intellectual disability (ID).^[1]^ To date, THOC6 pathogenic variants have been observed in patients with moderate to severe ID, accompanied by facial dysmorphic features and severe congenital malformations.^[6,7]^

## 2. Case presentation

A 35-day-old Palestinian infant was brought to the hospital by family members complaining of loud breathing sounds and a cough. Antenatally, the mother received regular follow-up care until the end of the second trimester. The patient was born to healthy parents who are first cousins, a G1P1A0 mother. The infant was delivered vaginally at 38 weeks gestation with a birth weight of 2.3 kg, a height of 45 cm, and a head circumference of 30 cm. However, Apgar score is not available. Postnatally, dysmorphic features, ambiguous genitalia, and anal atresia with a perineal fistula were noted; pelvic ultrasound showed bilateral inguinal gonads with male internal organs, and an echocardiogram revealed 3 cardiac defects: ventricular septal defect (VSD), patent foramen ovale (PFO), and peripheral pulmonary stenosis (PPS). Following evaluation, the patient underwent surgical correction for an anorectal malformation and was discharged 3 days later.

**Figure 1.**
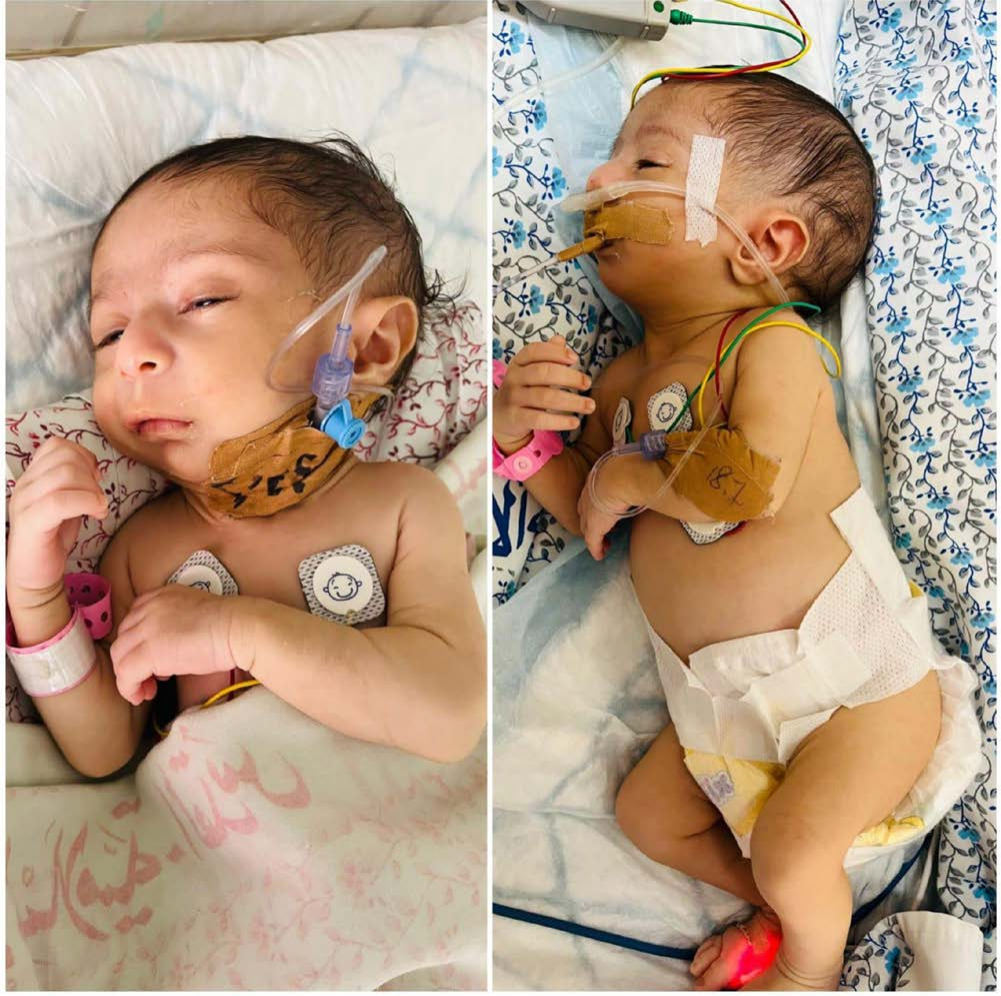
Patient photographs showing several dysmorphic fascial features.

Physical examination: The patient’s weight, height, and head circumference were 2.7 kg (−3.2SD), 47 cm (−3SD), and 32 cm (−3SD), respectively. Dysmorphic facial features were observed, including a prominent hairy forehead, long philtrum, microretrognathia, triangular face, small tongue, prominent right ear, posteriorly rotated ears, and bilateral epicanthal folds (Fig. 1). Respiratory examination revealed rapid breathing and bilateral wheezing, with a chest X-ray suggesting a chest infection.

Neurological examination showed normal peripheral tone, axial hypotonia, good Moro reflex, and good sucking. Genitourinary examination confirmed ambiguous genitalia, characterized by a 1 cm phallus with hypospadias and fused labioscrotal folds, with palpable right gonad, prompting the need for additional investigations. The remainder of the physical examination was unremarkable. Chromosomal analysis revealed a normal male karyotype (46, XY). Subsequent laboratory t tests were conducted and result summarizes in Table 1. The microdeletion on the Y chromosome was suspected, and DNA analysis by polymerase chain reaction of the azoospermia factor, zinc finger protein, Y-linked, and sex-determining region Y regions was performed. The test results showed none of the tested deletions were found (Table 2).

**Table 1:** Laboratory results.

Y chromosome microdeletion	Region AZF a		Region AZF b		Region AZF c		Region ZFY	Region ZRY
	sY84	sY86	sY127	sY134	sY254	sY255		
Tested	X	X	X	X	X	X	X	X
Result	Present	Present	Present	Present	Present	Present	Present	Present

No deletions were identified in the tested regions of the Y chromosome.

AZF = azoospermia factor, ZFY = zinc finger protein, Y-linked, SRY = sex-determining region Y.

**Table 2:** DNA analysis of chromosome Y revealed no deletions in any tested region.

No	Gene	Transcript	Position	Nucleotide	Amino acid	Genom AD	dbSNP	Zygosity	ACMG classification
1	THOC6	NM-024339.5	16:3075825	c.155+1G>T	–	–	–	HOM	Likely pathogenic

ACMG = American College of Medical Genetics and Genomics, dbSNP = database of single nucleotide polymorphisms, DNA = deoxyribonucleic acid, THOC6 = THO complex subunit 6.

This complex clinical presentation with wide genetic differential diagnoses, including Seckel syndrome, Rubinstein–Taybi syndrome, Bowen–Conradi syndrome, Acrocallosal syndrome, and Mowat–Wilson syndrome, prompted extensive genetic testing. Whole exome sequencing conducted at Hadassah Medical Organization identified the THOC6 (c.155+1G>T) variant mutation, revealing a homozygous G->T intron alteration at the splice site donor location (chr16:3075825). This mutation is predicted to lead to abnormal protein translation of the THCO6 protein, resulting in the diagnosis of BBIS (Table 3). The genomic amplification of axon 3 of the THOC6 gene and direct sequencing for father and mother were performed, which showed that both parents are heterozygous for the variant c.155+1G>T in intron 3 of the THOC6 gene.

**Table 3:** The exon sequence shows a c.155+1G>T variant mutation in the THOC6 gene.

Parameter	Measured value	Reference range
FSH	0.46 mlU/mL	0.95–11.95 mlU/mL
LH	0.18 mlU/mL	0.75–12.7 mlU/mL
TSH	3.99 mlU/mL	0.35–4.94 mlU/mL
T4	0.84 mlU/mL	0.70–1.84 mlU/mL
Androstenedione	0.3 ng/mL	0.4–1.5 ng/mL
Total Testosterone	4.87 ng/dL	262–870 ng/dL
Cortisol (baseline)	7.24 μg/dL	5–25 μg/dL
Cortisol (60 min)	48 μg/dL	18–64 μg/dL
17-OH progesterone	23 nmol/L	0.2–6.5 nmol/L

17-OH progesterone = 17-Hydroxyprogesterone, FSH = follicle-stimulating hormone, LH = luteinizing hormone, TSH = thyroid-stimulating hormone.

## 3. Discussion

BBIS is a rare neurodevelopmental condition inherited in an autosomal recessive manner and associated with pathogenic variants in the THOC6 gene.^[8]^ This neurodevelopmental disorder has a core clinical feature that includes delays in development, varying degrees of ID and dysmorphic facial features. Additionally, individuals may exhibit other abnormalities such as unusual microcephaly, as well as heart and kidney defects, hydrocephalus, multiple skeletal anomalies, and hyper-gonadotropic hypogonadism. In males, there may also be cryptorchidism.^[1,6]^ Facial features can vary and may include a tall forehead, short eyes that slant upward, sometimes with deep-set eyes, and a long nose with a prominent columella.^[7]^

Our case represents a 35-day-old infant who was admitted to the hospital by his family to inquire about his respiratory symptoms. Interestingly, the patient underwent surgical correction for anal atresia with a perineal fistula. The physical examination revealed dysmorphic facial features and ambiguous genitalia. A pelvic ultrasound revealed bilateral gonads in the inguinal area with male internal organs, and an echocardiogram showed multiple cardiac defects, including VSD, PFO, and PPS. Further tests, including karyotyping, an adrenocorticotropic hormone stimulation test, and hormone level measurements, indicated abnormal hormone levels and suspected a microdeletion on the Y chromosome. However, polymerase chain reaction amplification dismissed this suspicion later. These findings, combined with abnormal growth development and parameters for his age, underscore the importance of an underlying issue affecting this infant.

Subsequent DNA analysis revealed THOC6 gene mutation, specifically the c.155+1G>T variant, which is associated with BBIS. According to the ClinVar-NCBI, the THOC6 c.155+1G>T variant is predicted to disrupt the GT donor site and interfere with normal splicing; this variant appears to be rare, as it has not been reported previously in the literature or in a large population database. Variants that disrupt the consensus splice donor site in THOC6 are expected to be pathogenic. Both parents were found to be carriers of this variant. It was recommended that the patient continue follow-up with various specialists, including pediatricians, endocrinologists, neurologists, cardiologists, and geneticists.

Some clinical features noted in this patient align with those commonly reported in BBIS. These include a prominent forehead, elongated philtrum, prominent right ear, microretrognathia, triangular facial shape, tiny tongue, and bilateral epicanthus folds. Additionally, cardiac anomalies such as VSD, PFO, and persistent pulmonary stenosis (PPS) are also already reported.^[6,7,9]^ On the other hand, our case exhibits uncommon clinical features such as posteriorly rotated ears, anorectal malformation, and ambiguous genitalia. To our knowledge, there have been no previously reported BBIS cases associated with ambiguous genitalia.

Differential diagnoses for THOC6 syndrome include Seckel syndrome (AR), which presents with growth restriction and facial features; Rubinstein–Taybi syndrome (AD), distinguished by broad thumbs and halluces; Bowen–Conradi syndrome (AR), marked by joint contractures and early mortality; acrocallosal syndrome (AR), which has limb anomalies and macrocephaly; and Mowat–Wilson syndrome (AD), characterized by Hirschsprung disease and distinctive facial features.^[2]^

The THOC6 gene is located in the 16p13.3 region of chromosomes (chr16:30,24,027–30,27,755, GRCh38/hg38), and it is a part of the THO complex and interacts with other components to create the TREX complex. This complex appears to have a dynamic structure that involves ATP-dependent remodeling.^[9]^

In other words, TREX is a complex made up of THO, which includes THOC1, 2, 5, 6, 7, and Tex1, along with other proteins that are remarkably conserved across diverse organisms. This complex plays a critical role in various cellular functions and homeostasis, such as gene expression, genome stability, and embryogenesis.^[10]^ Specifically, the THO complex plays a crucial role in stem cell renewal, neuronal differentiation, brain synapse development, and dopamine neuron survival, which may explain the neurological manifestations observed in our patient.^[2]^

Recent studies have linked mutations in the THOC6 gene, which is crucial for RNA processing and mRNA export, to clinical features such as cryptorchidism and hypogonadism. These mutations disrupt the THO complex, impairing its function and leading to the mislocalization of the protein from the nucleus to the cytoplasm. This dysfunction in mRNA processing affects the hypothalamic-pituitary-gonadal axis, resulting in gonadal insufficiency and abnormal testicular descent. Additionally, the mutation increases apoptosis in cultured cells, further highlighting THOC6’s critical role in male reproductive development.^[11]^

Specific mutations in the THOC gene are associated with some disorders. For example, hemizygous missense mutations in THOC2 have been linked to syndromic X-linked ID. Additionally, a translocation disrupting THOC2 has been reported in a case involving cognitive impairment and cerebellar hypoplasia.^[6]^

Elizabeth A. Werren and colleagues used both in vivo (mouse) and in vitro (human stem cell-derived) models to expand the known spectrum of **THOC6 variants** linked to **THOC6 intellectual disability syndrome**. They showed that these variants cause disease through a **loss-of-function mechanism**. Although global **mRNA export** via the TREX dimer remains unaffected, disruption of the **THOC6-dependent TREX tetramer** impairs mRNA processing. This defect leads to abnormal **proliferation and differentiation** of **neural progenitor cells** into excitatory neurons in human stem cell models. In mice, complete loss of THOC6 results in **embryonic lethality**, highlighting species-specific differences. Overall, the study reveals a broader role for the **TREX tetramer** in **RNA processing** and **neural development**.^[12]^

THOC6 exhibits broad expression during the early stages of development and plays a crucial role in embryogenesis. This wide-ranging function may help explain why patients with BBIS present with various congenital anomalies. This wide range of clinical features, especially nonspecific ones, makes it a challenge for doctors to recognize; consequently, further reports will assist in refining the spectrum of clinical features associated with this syndrome.

## 4. Limitation

A significant limitation of this study is the inability to include photographic documentation of the infant’s ambiguous genitalia, which could have enhanced the clarity of the clinical findings and provided a more comprehensive visual reference for understanding the presentation of the condition. Unfortunately, the authors were unable to obtain a photograph of the baby’s genitalia due to the parents’ refusal to allow imaging for this purpose.

## 5. Conclusion

This case highlights the importance of a comprehensive diagnostic approach in rare syndromes like BBIS, especially when ambiguous genitalia and congenital anomalies are present. Genetic testing, such as **THOC6** gene sequencing, is essential for confirming the diagnosis. Future studies should focus on the functional implications of the **c.155+1G>T** variant using in vivo and in vitro models, exploring its impact on mRNA processing and export, particularly in relation to ambiguous genitalia and sexual development disorders. Additionally, research should investigate long-term clinical outcomes and expand the phenotypic spectrum of BBIS, which will aid in the development of potential therapeutic interventions.

## Acknowledgments

We would like to thank the patient’s family for cooperating in this study and the research unit in the College of Medicine at Hebron University for their continuous support.

## Author contributions

**Data curation:** Mousa Humeedat.

**Resources:** Nedal Manasra.

**Supervision:** Baraa Maraqa

**Validation:** Maaweya Jabareen.

**Writing – original draft:** Mousa Humeedat, Maaweya Jabareen, Husein Sarahenh, Nedal Manasra.

**Writing – review & editing:** Husein Sarahenh.
